# Autonomic deficit not the cause of death in West Nile virus neurological disease

**DOI:** 10.1007/s10286-013-0213-y

**Published:** 2013-10-25

**Authors:** Hong Wang, Venkatraman Siddharthan, Jeffery O. Hall, John D. Morrey

**Affiliations:** 1 Department of Animal, Dairy, and Veterinary Sciences, School of Veterinary Medicine, Institute for Antiviral Research, Utah State University, 4700 Old Main Hill, Logan, UT 84322-4700 USA; 2 Department of Animal, Dairy, and Veterinary Sciences, Utah Veterinary Diagnostic Laboratory, School of Veterinary Medicine, Utah State University, Logan, UT 84322-5700 USA

**Keywords:** West Nile virus, Autonomic, Parasympathetic, Heart rate variability

## Abstract

**Introduction:**

Some West Nile virus (WNV)-infected patients have been reported to manifest disease signs consistent with autonomic dysfunction. Moreover, WNV infection in hamsters causes reduced electromyography amplitudes of the gastrointestinal tract and diaphragm, and they have reduced heart rate variability (HRV), a read-out for the parasympathetic autonomic function.

**Methods:**

HRV was measured in both hamsters and mice using radiotelemetry to identify autonomic deficits. To identify areas of WNV infection within the medulla oblongata mapping to the dorsal motor nucleus of vagus (DMNV) and the nucleus ambiguus (NA), fluorogold dye was injected into the cervical trunk of the vagus nerve of hamsters. As a measurement of the loss of parasympathetic function, tachycardia was monitored contiguously over the time course of the disease.

**Results:**

Decrease of HRV did not occur in all animals that died, which is not consistent with autonomic function being the mechanism of death. Fluorogold-stained cells in the DMNV were not stained for WNV envelope protein. Fourteen percent of WNV-stained cells were co-localized with fluorogold-stained cells in the NA. These data, however, did not suggest a fatal loss of autonomic functions because tachycardia was not observed in WNV-infected hamsters.

**Conclusion:**

Parasympathetic autonomic function deficit was not a likely mechanism of death in WNV-infected rodents and possibly in human patients with fatal WN neurological disease.

## Introduction

We have previously reported that autonomic nervous system function deficits can be observed in hamsters infected with WNV [32]. This is consistent with human clinical studies and case reports documenting WNV disease signs such as respiratory distress [29], diaphragmatic paralysis [2], gastrointestinal involvement, urinary retention [31], bladder dysfunction [29], fatigue [19], and cardiac arrhythmia [4]. West Nile virus infection is fatal in <1 % of human cases [20], but the fatality rate in experimentally infected mice and hamsters is typically reported to be between 20 and 80 % [23, 28, 35]. Therefore, rodents provided an opportunity to ask questions about the mechanism of death from WN neurological disease.

In WNV-hamster studies, autonomic function was suppressed as measured by reduced heart rate variability (HRV), which is a well-accepted read-out for monitoring sympathetic and parasympathetic autonomic functions [11, 14]. The major influence of the parasympathetic HRV is cardiopulmonary coupling, which is the neurological connectivity that causes the heart rate to increase during resting respiratory inspiration and decrease during expiration, which is physiologically referred to as respiratory sinus arrhythmia [12]. This autonomic process results in efficient cardiopulmonary function. Therefore, healthy autonomic parasympathetic function results in higher HRV. Conversely, unhealthy parasympathetic function results in reduced HRV. The parasympathetic input for cardiopulmonary coupling or sinus arrhythmia occurs through the cervical trunk of the vagus nerve innervated by neurons in the DMNV and NA of the medulla [34]. If DMNV or NA neurons were damaged or their numbers were reduced, the sympathetic response would be dominant. Reduced parasympathetic and increased sympathetic responses typically result in tachyarrhythmia, and if severe enough, cardiac arrest [13]. Therefore, our approach to measure the effects of WNV on the parasympathetic cardiopulmonary coupling was to measure HRV, infection of the DMNV and NA neurons, and tachyarrhythmia in infected rodents.

We have recently determined that respiratory insufficiency is highly correlated with mortality in WNV-infected hamsters, and that respiratory insufficiency was due to neurological disease [26, 33]. This respiratory insufficiency could be due to the neurodegeneration of neurons controlling respiratory function including the raphe obscurus [8], retrotrapezoid nucleus and parafacial respiratory group [1, 10], rostral ventrolateral medulla [15], or pre-Bötzinger complex [3] in the brainstem, phrenic motor neurons in the cervical cord, or in the posterior hypothalamus [18]. Even though WNV suppresses respiratory function, we did not know specifically if autonomic dysfunction correlated to respiratory insufficiency or mortality, which was the subject of this study.

## Materials and methods

### Ethical statement

This study was conducted in accordance with the approval of the Institutional Animal Care and Use Committee of Utah State University (IACUC approval #1079 and #1488; WNV APHIS Permit # 47210). The work was done in the AAALAC-accredited Laboratory Animal Research Center of Utah State University and in accordance with the National Institutes of Health Guide for the Care and Use of Laboratory Animals.

### Animals and viruses

Adult female Syrian golden hamsters or C57BL/6 mice >7 weeks of age were used (Charles River Laboratories, Wilmington, MA). Animals were randomized to treatment groups. Rodents were judged as moribund if they did not step forward if prodded, or if they did not right themselves when placed on their backs. WNV was diluted in minimal essential medium immediately prior to subcutaneous (s.c.) injection in the groin area [24, 25, 27]. Hamsters were injected with a volume of 0.1 mL containing 5.7 × 10^7^ pfu of a New York WNV isolate from crow brain [16, 17]. Mice were injected with a volume of 0.1 mL containing 2.5 × 10^6^ pfu of a WN02 isolate designated as Kern 515 from Dr. Robert Tesh (Mosquito, 10/05/07, Kern County, CA, TVP 10799 BBRC lot # WNVKERN515-01, University of Texas Medical Branch Arbovirus Reference Collection). These two strains of WNV were used because of suitable mortality in the two rodent strains used and to demonstrate that the findings of this report are not rodent- or virus strain specific.

### Heart rate variability (HRV) analysis

Radio telemetry devices (TA10EEAT-F20, Data Sciences International, New Brighton, MN) were surgically implanted in a subcutaneous pocket [21, 30, 32]. Two recording-leads were subcutaneously tunneled toward the left and right clavicular region where the tips of leads were sutured to the pectoral muscles. The DSI ETA-F20 transmitter transmitted ECG tracings with a sampling rate of 1,000 Hz as an analog radio signal with a digital signature. The receivers, under the cages, were connected to a data acquisition matrix hard-wired to a PC-based computer running Dataquest A.R.T. Silver System software (Data Science International, DSI). The duration of each reading was 2 min and one reading was obtained every hour. The acquisition software was IOX Base 8c software, and the ECG analysis software was ECG auto with HRV + module to generate HRV data including the Poincaré plot (emka Technologies, USA, Falls Church, VA). The acquired data from the DSI software were converted to data suitable for IOX software analysis by using ECG Auto 3.1 (emka Technologies, USA, Falls Church, VA). The times of the resting status of the hamsters were recorded multiple times each day. Data from resting hamsters, as opposed to alert moving hamsters, were used to minimize background noise from movement of the animals.

The Poncairé plot is constructed by plotting each R to R (RR) interval in milliseconds (ms) on the *x*-axis, and the previous adjacent RR interval on the *y*-axis [5]. The more rapid the heart rate, the smaller is the RR interval. Conversely, the slower the heart rate, the larger is the RR interval. Since the heart rate is neurologically coupled to the inspiration and expiration of the lung, referred to as cardiopulmonary coupling, the RR intervals will be different than the previous RR intervals as the animal breathes. This cardiopulmonary coupling is controlled by autonomic function. If the autonomic function is healthy, the individual dots on the Poincaré plot will be widely distributed in a comet shape. As autonomic function is reduced, the comet shape will condense to a small shape or dot [5]. Data sample times were 2 min for hamsters and 5 min for C57BL/6 mice. The Poincaré data were quantified as nonlinear measurements of HRV by measuring the standard deviations in the perpendicular direction of the pattern (SD1) and the SD of the parallel direction of the data pattern (SD2) using the Kubios HRV Analysis software (version 21.).

### Radiotelemetry for temperature, heart rate, and activity

The same radio telemetry chips and surgical procedures used for HRV analysis were used. Data were obtained continuously throughout the course of the experiment. The data were graphed using GraphPad Prism 5.0.

### Fluorogold and WNV envelope staining

DMNV and NA regulate considerable parasympathetic autonomic function to the heart through the cervical trunk of the vagus nerve [6]. To fluorescently locate the DMNV and NA, the cervical trunk of the vagus nerve was injected with 1 μL of 2 mg/mL of fluorogold. The tissues were fixed for histological analysis 3 days after fluorogold injection. The hamsters were injected with euthasol (100 mg/kg, i.p) and cardiac perfused (i.v.) with 0.01 M PBS followed by 4 % paraformaldehyde. The brainstems maintained in 30 % sucrose overnight were cut into coronal sections (10 μm) by a cryostat instrument (Leica CM 1900). They were permeabilized in 0.5 % triton X-100, blocked with 5 % goat serum in 0.1 % BSA, incubated with 7H2 anti-WNV mouse mAb (1:450, BioReliance, Invitrogen Bioservices, Rockville, MD) and rabbit anti-fluorogold polyclonal antibody (1:3000, Fluorochrome, LLC, Denver, Colorado) at 4 °C overnight. The sections were then incubated with Alexa-fluor^®^ 568 goat anti-mouse IgG antibody and Alexa-fluor^®^ 488 goat anti-rabbit IgG antibody (Life Technologies, Grand Island, NY). Slides were examined using a Carl Zeiss Axioskop-2 microscope (Carl Zeiss Microscopy, LLC, Thornwood, NY) and X-Cite fluorescence illumination (Ontario, Canada), connected with CRI-Nuance (Grantham, UK) multispectral image system.

## Results

**Fig. 1:**
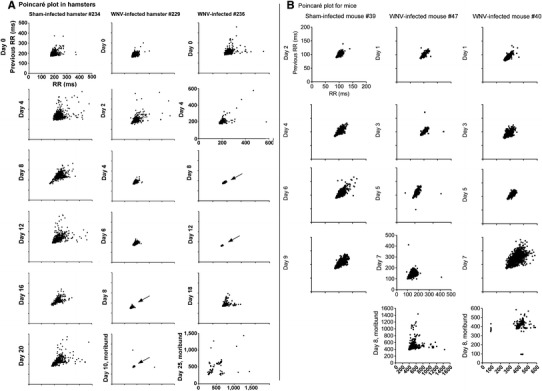
Poincaré plots to detect parasympathetic autonomic function over the course of disease in **a** hamsters and **b** mice injected subcutaneously with WNV. Data from representative animals and days after viral challenge are shown from a total of **a** five sham-infected hamsters, six WNV-infected hamsters, **b** four sham-infected mice, and five WNV-infected mice. Unlabeled axes have the same values as listed in the first upper left graph for **a** hamsters and **b** mice. *Arrows* indicate loss of HRV

All six WNV-infected hamsters lost some autonomic function during the course of the disease as evidenced by the Poincaré plots, wherein two representative WNV-infected hamsters compared to a sham-infected hamster are shown (Fig. 1a). As explained in “Materials and methods”, the autonomic HRV is healthy if the dots are widely distributed. If the function or numbers of parasympathetic autonomic neurons are diminished, the HRV is diminished and the dots condense to a smaller shape. Hamsters displayed patterns of diminished autonomic nervous function during the course of infection as illustrated with hamsters #229 and #236, which became moribund on days 10 and 25, respectively. The condensed data pattern of hamster #229 continued until the day of termination, whereas the condensed patterns of hamster #236 disappeared later in the course of disease as illustrated on day 18 (Fig. 1a). On the day of termination at day 25, the distribution of data points expanded even more. Overall, condensed data point patterns characteristic of autonomic dysfunction from WNV-infected hamsters did not consistently correlate with the time of mortality (data of all animals not shown). None of the sham-infected hamsters possessed condensed data patterns, as illustrated with hamster #234 (Fig. 1a).

The HRV of each hamster was quantified from the Poincaré data by measuring the standard deviation (SD) in the perpendicular direction of the pattern (SD1) and the SD of the parallel direction of the data pattern (SD2) (Kubios HRV Analysis, version 2.1). The smaller numbers of SD1 and SD2 indicated smaller heart rate variability. Two SD below the mean of sham-infected animals was used as a cutoff for normal values, i.e., values below 2SD were considered abnormal (Table 2). The HRV from animals #227, 229, 236, and #238 appeared to be low on days 8 and 12 after WNV challenge. Some of the values from these hamsters were below the cutoff of 2SD. One hamster (#238) gained higher HRV values before succumbing to the disease. The remainder of the hamsters (#239 and #240) did not develop low HRV according to this quantitative analysis, but still succumbed to the disease.

Poincaré plots were generated for WNV- and sham-infected C57BL/6 mice (Fig. 1b). Unlike the hamsters, condensed data point patterns did not develop in nearly all of the mice with fatal WNV infection. Mouse #44 and mouse #50 developed values below 2SD of the sham-infected means, but the low HRV values were not associated with the time of mortality (Table 1). Conversely, the distribution of data points often expanded during the course of disease, which supported the observation in hamster that patterns characteristic of parasympathetic autonomic dysfunction did not correlate with mortality.

**Table 1:** Quantification of heart rate variability from Poincaré plot data in WNV-infected rodents using Kubios HRV Analysis software (version 21)

Hamsters												
Animal #	Death day	Day 0^A^	Day 4	Day 8	Day 12	Day 14	Day 15	Day 16	Day 18	Day 19	Day 20	Day 25
#227	9	20, 33	8.4, 13	11, 11								
#229	14	42, 57	21, 25	8.0, 10^B^	6.8^B^, 7.8^B^							
#236	18	101, 107	66, 74	8.4, 9.4^B^	8.2, 8.8^B^							
#238	19	37, 43	26, 31	64, 89	10.8, 6.6^B^			82, 120		134, 143		
#239	25	22, 41	11, 17	31, 36	75, 84	91, 103			58, 76		48, 62	126, 125
#240	16	28, 40	20, 33	58, 68	33, 35	30, 31	16, 17					

Mice								
Animal #	Death day	Day 1	Day 3	Day 5	Day 7	Day 8	Day 9	Day 10
#40	8	7.1, 9.8	9.6, 16	5.6, 11	19, 21	56, 86		
#42	8	4.1, 9.1	3.6, 6.8	7.3, 12	4.3, 10	26, 37		
#44	8	2.5^C^, 4.3	1.7, 6.1	8.3, 12	8.1, 8.0	21, 47		
#47	8	3.9, 4.8	6.9, 11	5.8, 12	12, 24	36, 68		
#50	10	4.2, 4.1	3.4, 5.7	3.3, 8.7	7.1, 12	4.2, 11	2.0^C^, 4.7	48, 90

The smaller numbers of SD1 and SD2^a^ indicated smaller heart rate variability. SD1 and SD2 are the standard deviations in the X1 (perpendicular direction) and X2 (parallel direction) to the scatter distribution of the Poincaré plot

^A^Days after WNV subcutaneous challenge

^B^Values considered abnormal when below two standard deviations of sham-infected hamsters, SD1, SD2 ≤7.8, 10.5

^C^Values considered abnormal when below two standard deviation of sham-infected mice, SD1, SD2 ≤2.6, 1.8

**Fig. 2:**
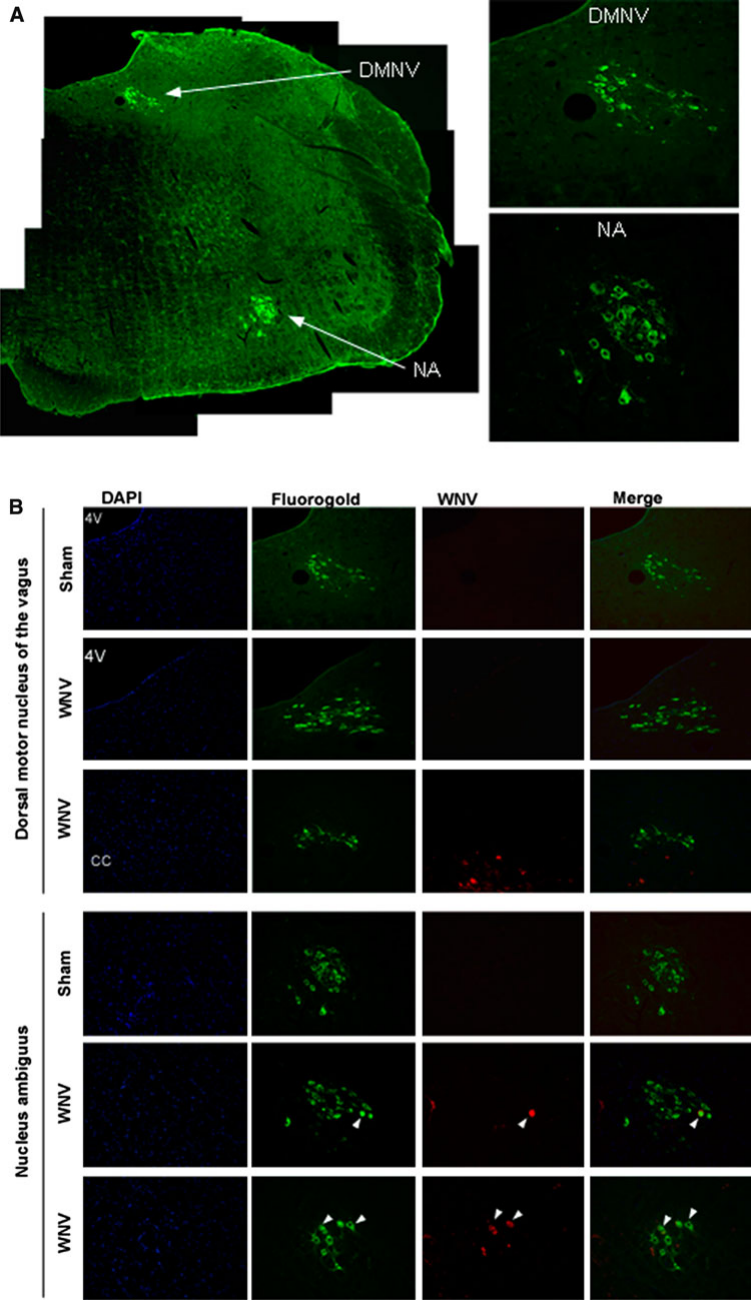
Staining of the DMNV and NA for WNV envelope antigen. **a** Five hamsters were injected into the cervical trunk of the vagus nerve with fluorogold. After 3 days, sections of the brains in the area of the DMNV and NA were prepared and stained for WNV envelope antigen. The fluorogold-stained areas were identified to be the DMNV and NA using a hamster stereotaxic atlas [22]. On the left of a representative hamster, images were assembled from the same brain section to construct a hemisphere identified to be the fluorogold staining of DMNV and NA. On the right, higher magnifications of the image of the DMNV (*upper*) and NA (*lower*) are shown. **b** Images of the DMNV and NA identified with fluorogold were stained for WNV envelope antigen in WNV- and sham-infected hamsters. The animals were injected s.c. with WNV or sham. Six days later they were injected into the cervical trunk of the vagus nerve with fluorogold, after which the brains were processed for staining for WNV envelope antigen and with DAPI to detect nuclear DNA (3 days after dye injection). *Arrows* identify WNV-positive cells

Since the parasympathetic function of the heart is primarily controlled by vagal nerve cells located in the medulla oblongata, we applied a previously published technique [6] to identify the location of vagal neuron cell bodies. The neuron cell bodies controlling parasympathetic innervation of the thorax and abdominal viscera were identified by injecting the cervical trunk of the vagus nerve with fluorogold dye. Fluorogold dye then transported through the axons into the respective neuron cell bodies of the DMNV and the NA. Positive cells stained with fluorogold could be visualized in coronal sections of the medulla oblongata using as references the fourth ventricle, central canal, and a hamster brain atlas [22] (Fig. 2a). The same technique was applied to hamsters injected s.c. with WNV to determine if WNV infects these cells (Fig. 2b). In a total of six hamsters, no WNV-infected cells co-localized with fluorogold in the DMNV (0 WNV-positive cells co-localized with 193 fluorogold-positive cells), whereas, 14 % of WNV-stained cells (11 WNV-positive cells co-localized with 80 fluorogold-positive cells) co-localized with fluorogold-stained cells in the NA (Table 2). These data suggest that parasympathetic autonomic function controlled by the DMNV were not affected by WNV infection, whereas the NA may have been affected by WNV.

**Table 2:** Co-localization of WNV-stained neurons with fluorogold-stained neurons in the dorsal motor nucleus of vagus (DMNV) and nucleus ambiguus (NA)

	WNV-positive staining co-localized with fluorogold-positive cells per total fluorogold-positive cells (%)	
	WNV-infected hamsters	Sham-infected hamsters
DMNV	0/193 (0 %)^A^	0/157 (0 %)^B^
NA	11/80 (14 %)^C^	0/35 (0 %)^D^

Criteria for killing and necropsy—no stepping when prodded

^A^Fifteen slides from six hamsters

^B^Seven slides from two hamsters

^C^Nine slides from four hamsters

^D^Four slides from one hamster

**Fig. 3:**
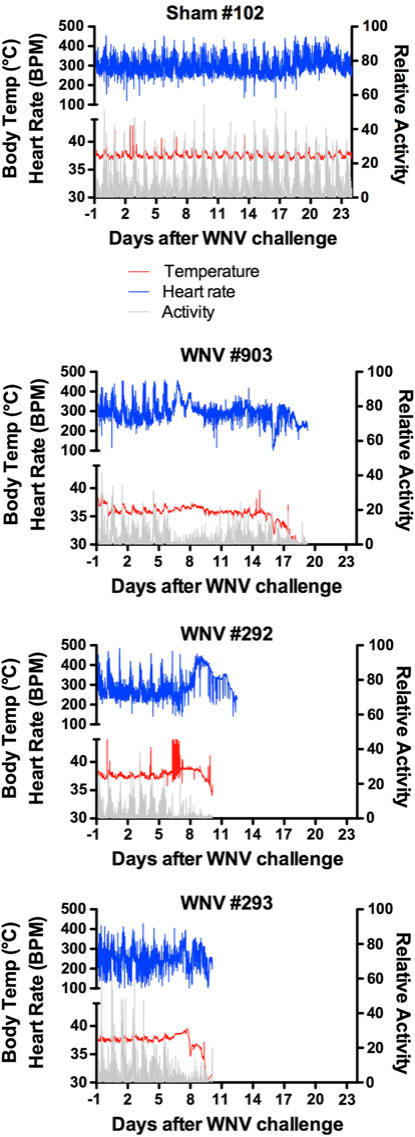
Heart rate (*blue*), body temperature (*red*), and movement activity (*gray*) measured by radio telemetry in hamsters challenged s.c. with sham or WNV. Data from representative animals are shown from a total of two sham-infected hamsters, 8 WNV-infected hamsters

To determine if infection of parasympathetic neurons affected cardiac parasympathetic function, tachycardia was investigated in WNV-infected hamsters using surgically implanted radio telemetry chips. These chips faithfully reported heart rate, in addition to body temperature and movement activity in two sham-infected control hamsters over 24 days, as illustrated with sham #102 (Fig. 3). Diurnal and nocturnal cycling was readily observed in these animals and underscored the need to record heart rate throughout the 24-h cycle. The data from three representative WNV-infected hamsters are shown in Fig. 3. Animals #903 and #293 showed no evidence of tachycardia. Animal #292 showed some evidence of possible tachycardia at about days 8–10. Nevertheless, tachycardia did not correlate with mortality, which suggested that loss of parasympathetic autonomic function was not the main mechanism of death.

## Discussion

The main contribution of this study is the evidence generated to show that loss of parasympathetic autonomic function does occur in hamsters, but is not the mechanism of death in WNV-infected rodents. Three physiological parameters provided this evidence, namely, HRV as represented in Poincaré plots, staining of the DMNV and NA for WNV envelope antigen, and measurement of tachycardia. In a previous publication by us [26] demonstrating reduced HRV in WNV-infected hamsters, standard time domain measurements (e.g., standard deviation of normal RR, root mean square of the differences between consecutive RR) and frequency domain measurements (low- and high-frequency power) were used. Subsequently in this report, we have used the Poincaré plots to visualize the HRV, and subjectively believe that it is more dependable and unambiguous in detecting reduced HRV in these WNV-rodent models. Moreover, the parasympathetic analysis of the current study differed from the prior study in that measurements were used when the animals were motionless and appeared to be sleeping. This reduced erroneous recordings due to movement of the electrodes or by muscle EMGs during times of activity. Clearly, some animals had loss of HRV on the last day of death as identified by condensed data points, but other animals did not have condensed data points on the day of death. Moreover, mice never did develop condensed data points during the course of disease. Therefore, loss of HRV could not have been the mechanism of WNV-induced death in rodents.

Autonomic function is divided into the ‘fight-or-flight’ sympathetic response and ‘rest-and-digest’ parasympathetic response. The neuron cell bodies innervating the thorax and abdominal viscera parasympathetic responses were identified by observing stained neurons in the DMNV and NA from animals injected in the cervical trunk of the vagus nerve with fluorogold dye [6]. No WNV-stained cells were observed in the DMNV, and a small number of cells were observed in the NA. The parasympathetic autonomic response alone was likely to not be the mechanism of WNV-induced death, because the vast majority of these cell bodies were not stained for WNV envelope antigen reflecting viral replication.

This hypothesis was supported by analysis of heart rate. If the viruses were to functionally damage neurons in the DMNV and NA, the cardiac parasympathetic response would be reduced and leave a dominant sympathetic response. This typically results in tachyarrhythmia, and if severe enough, cardiac arrest [13]. Since hamsters manifest strong circadian rhythm [7], single measurements of heart rates during the day may result in misidentification of tachyarrhythmias. To overcome this problem, the heart rate was monitored continuously over the course of the experiment using radio telemetry chips. As expected, strong fluctuations of diurnal and nocturnal rhythms were observed prior to development of neurological disease. There was no consistent tachycardia observed, however, during the development of fatal disease. Moreover, in a recent study, cardiac arrhythmia did not correlate with mortality [26]. Therefore, tachyarrhythmia did not consistently occur in lethal WNV disease in hamsters.

An interesting occurrence can be observed in Fig. 3 where the circadian rhythm of a particular hamster #903 appeared to be lost beginning at day 8 after WNV challenge. Other hamsters, but not all, that recover from WNV infection manifest this loss of circadian rhythm (data not shown), which may be attributed to loss of higher-order functions in areas such as the suprachiasmatic nucleus [7]. The cause of loss of circadian rhythm in WNV-infected rodents and how this might relate to sleep disorders or fatigue in WNV patients await further investigations.

Another interesting observation seen qualitatively in Fig. 1 and quantitatively in Table 1 is the increase in HRV in some hamsters (#238, #239) and in all mice just before death. Even though cardiopulmonary coupling is largely responsible for high-frequency rhythms, low-frequency rhythms controlled by higher brain structures might also dominate during WNV infection and could conceivably be responsible for increased HRV. Moreover, the differences in cytokine responses that may occur between individual animals and between species may contribute to differences in HRV due to viral infection [9]. These issues should be investigated in future studies.

The results of this study augmented the finding that respiratory insufficiency correlated strongly with the mortality of rodents infected with WNV and were the likely the predominant mechanism of death. Gliosis, paravascular cuffing, neuronal phagia, and WNV-specific staining were observed in the rostral ventrolateral medulla controlling respiratory motor functions of hamsters with respiratory insufficiency as measured by plethysmography, but little staining was observed in the DMNV or NA. These prior studies [26, 33] and the current study suggest that respiratory insufficiency, not loss of parasympathetic autonomic function, is the primary mechanism of death in WNV-infected rodents and possibly human patients.

In this work we showed that WNV infection of rodents may or may not cause autonomic function deficits as measured by HRV depending on the species, hamsters and mice, yet both species succumb to infection. The lack of tachycardia and WNV infection in the dorsal motor nucleus of vagus or nucleus ambiguous controlling parasympathetic functions also suggest that autonomic dysfunction in rodents and possibly human patients is not likely to be the physiological mechanism of death, despite the clinical observation that disease signs and symptoms may reflect some degree of autonomic function deficits.
